# Tumor lysis syndrome in breast cancer: a systematic review of the literature

**DOI:** 10.3389/fonc.2026.1937565

**Published:** 2026-10-14

**Authors:** Min Zhang, Junli Li, Linhan Zeng, Yao Chen, Xuewu Huang

**Affiliations:** 1 The First Clinical Medical College, Guangzhou University of Chinese Medicine, Guangzhou, China; 2 The First Affiliated Hospital of Guangzhou University of Chinese Medicine, Guangzhou, China; 3 Guangdong Provincial Institute of Traditional Chinese Medicine Clinical Research, Guangzhou, China

**Keywords:** tumor lysis syndrome, breast cancer, aromatase inhibitors, endocrine therapy, case reports, systematic review

## Abstract

**Background:**

Tumor lysis syndrome (TLS) is a life-threatening oncologic emergency well-characterized in hematologic malignancies but considered rare in solid tumors. In breast cancer, TLS has been documented only in sporadic case reports, and the evolving treatment landscape—including CDK4/6 inhibitors, PI3K inhibitors, novel endocrine agents, and antibody-drug conjugates—may introduce previously unrecognized TLS risks. The only prior review on this topic was published in 2021 and has been superseded by numerous new cases.

**Methods:**

We systematically searched PubMed, Embase, and Web of Science from January 2000 to June 2026 for case reports and case series describing TLS in patients with breast cancer, following PRISMA guidelines. TLS diagnosis was verified against Cairo-Bishop criteria. Data on patient demographics, tumor characteristics, precipitating factors, laboratory abnormalities, management, and outcomes were extracted and analyzed descriptively.

**Results:**

Twenty cases of TLS from 20 publications were identified, with one additional case of successful TLS prevention. All patients had stage IIB–IV disease, with the majority (90%, 18/20) having stage IV disease; liver metastases were present in 55% (11/20). Precipitating factors included endocrine therapy (n=4, 75% mortality), HER2-targeted therapy (n=4, 50% mortality), chemotherapy (n=6, 50% mortality), radiotherapy (n=2, 50% mortality), PI3K inhibitor (n=1, survived), immunotherapy-chemotherapy combination (n=1, survived), and spontaneous TLS (n=2, 50% mortality). Overall TLS-related mortality was 50% (10/20). Endocrine therapy-associated TLS—predominantly aromatase inhibitors—carried the highest mortality (75%, 3/4). The only surviving patient in this group achieved long-term remission following supportive management.

**Conclusions:**

TLS in breast cancer, though rare, carries a high mortality rate approaching 50%. Notably, endocrine therapy—particularly aromatase inhibitors—emerges as a previously underappreciated TLS trigger with disproportionately poor outcomes. With the expanding use of novel targeted and endocrine agents in advanced breast cancer, clinicians should maintain heightened vigilance for TLS, especially in patients with high tumor burden and hepatic metastases. Early recognition and prompt intervention are essential to improve outcomes.

**Systematic Review Registration:**

https://www.crd.york.ac.uk/PROSPERO/, identifier CRD420261429658.

## 1 Introduction

Tumor lysis syndrome (TLS) is an oncologic emergency characterized by the rapid release of intracellular contents—including uric acid, potassium, phosphorus, and nucleic acids—into the systemic circulation following massive tumor cell breakdown. These metabolic derangements can precipitate acute kidney injury, cardiac arrhythmias, seizures, and death if not promptly recognized and managed. TLS has been well-characterized in hematologic malignancies with high proliferative rates and chemosensitivity, particularly Burkitt lymphoma and acute lymphoblastic leukemia, where prophylactic protocols have substantially reduced its incidence and associated mortality (1, 2).

In solid tumors, TLS has traditionally been considered a rare event. The slower doubling times, lower growth fractions, and more modest response kinetics of most solid malignancies were thought to confer relative protection against the massive cytolysis that characterizes TLS. However, over the past two decades, accumulating case reports have documented TLS across diverse solid tumor types (3, 4), particularly in patients with bulky disease, extensive metastatic burden, or hepatic involvement.

Breast cancer is the most commonly diagnosed malignancy (5) and the leading cause of cancer-related death among women globally. Historically, breast cancer has been regarded as low-risk for TLS, with only sporadic case reports appearing in the literature prior to 2010 (6). The prevailing view held that the modest tumor burden typical of most breast cancer presentations, combined with the relatively gradual cytoreduction associated with conventional chemotherapy, mitigated TLS risk. Early case reports of TLS in breast cancer date back to the 1980s and 1990s, primarily associated with tamoxifen or conventional chemotherapy (7, 8). These earlier reports were identified through handsearching and are cited here for historical context; they were not part of the systematic database search, which was limited to publications from 2000 onward.

This perspective, however, may no longer be tenable. The therapeutic landscape for breast cancer has undergone a paradigm shift over the past decade. Beyond conventional cytotoxic chemotherapy, the armamentarium now includes CDK4/6 inhibitors (palbociclib, ribociclib, abemaciclib), PI3K inhibitors (alpelisib) (9), antibody-drug conjugates (trastuzumab deruxtecan, sacituzumab govitecan), immune checkpoint inhibitors (10), and novel endocrine agents. These therapies offer unprecedented efficacy, particularly in patients with advanced or metastatic disease with high tumor burden—a population inherently predisposed to TLS. Recent case reports have implicated several of these novel agents as potential TLS triggers, including alpelisib (11), atezolizumab (12), and aromatase inhibitors such as letrozole and anastrozole (13, 14).

To date, the literature on TLS in breast cancer remains fragmented. A single brief review published in 2021 provided an initial synthesis, identifying a limited number of cases predominantly associated with chemotherapy and HER2-targeted therapy (15). Since then, a substantial number of new cases have emerged, more than doubling the published literature. These recent reports include cases triggered by contemporary therapeutic modalities—including PI3K inhibitors, immunotherapy combinations, and aromatase inhibitors—that were not previously represented. Moreover, they provide new insights into risk factors, diagnostic challenges, and management strategies with direct clinical implications.

This systematic review aims to provide a comprehensive, up-to-date synthesis of all published case reports of TLS in breast cancer from 2000 to 2026. Specifically, we seek to: [1]characterize the clinical presentation and precipitating factors; [2]evaluate diagnostic approaches, management strategies, and clinical outcomes; [3] identify risk factors that may inform clinical prediction; and [4] highlight the emerging TLS risk associated with novel therapeutic modalities. In doing so, we hope to raise clinical awareness of this rare but potentially fatal complication and provide a foundation for evidence-based prevention and management strategies in the modern treatment era.

## 2 Methods

### 2.1 Protocol and registration

This systematic review was conducted in accordance with the Preferred Reporting Items for Systematic Reviews and Meta-Analyses (PRISMA) 2020 guidelines (16). The protocol was prospectively registered with the International Prospective Register of Systematic Reviews (PROSPERO, registration number: CRD420261429658).

### 2.2 Search strategy

We systematically searched PubMed, Embase, and Web of Science from January 1, 2000, to June 25, 2026. The start date was chosen to cover the period following the first reported case of TLS in breast cancer (17). PubMed and Embase were selected as the core biomedical databases, and Web of Science was included for its multidisciplinary coverage and citation tracking capability. This three-database combination is consistent with the search strategies used in prior systematic reviews on TLS in solid tumors. The search strategy combined terms for TLS and breast cancer using Boolean operators, adapted for each database. For PubMed, we used the MeSH terms and title/abstract fields; for Embase, we used Emtree terms and title/abstract fields; for Web of Science, we used topic field searches. All database searches were limited to English language publications and case reports or reviews, with publication years between 2000 and 2026. One additional non-English publication identified through handsearching was included based on its English abstract. In addition to the database search, we performed handsearching of reference lists of relevant reviews and included articles, as well as targeted searches of individual journals and author names, to identify additional case reports not captured by the electronic search.The full search strategies for all databases are provided in the **Supplementary Table S1**.

### 2.3 Eligibility criteria

Studies were included if they met the following criteria: [1] patients with a confirmed pathological diagnosis of breast cancer (any subtype, any stage); [2] TLS diagnosed according to Cairo-Bishop criteria or as explicitly defined by the authors using standardized laboratory thresholds; [3] TLS occurring spontaneously or following any therapeutic intervention (chemotherapy, targeted therapy, endocrine therapy, radiotherapy, or combination regimens); [4] case reports or case series published in English between 2000 and 2026. One non-English publication Kawaguchi Ushio et al., (18) was included based on the detailed clinical data available in its English abstract, with TLS diagnosis and key clinical parameters verified from the abstract. Studies were excluded if they: [1] involved malignancies other than breast cancer as the primary diagnosis; [2] did not meet TLS diagnostic criteria; [3] lacked individual patient-level data; [4]were conference abstracts, editorials, reviews, or commentaries without original case data. One non-English publication Kawaguchi Ushio et al., (18) was included based on detailed clinical data available in its English abstract; the TLS diagnosis and key clinical parameters were verified from the abstract, and full-text translation was not required for data extraction.

### 2.4 Study selection and data extraction

Two reviewers independently screened titles and abstracts, followed by full-text assessment for eligibility. Disagreements were resolved through discussion. Data were extracted into a standardized Excel spreadsheet using a predefined template. Extracted variables included: first author, year of publication, patient age and sex, breast cancer subtype, stage, metastatic sites, TLS precipitant (chemotherapy/targeted/endocrine/radiotherapy/spontaneous), specific drugs administered, TLS diagnostic criteria (Cairo-Bishop or other), laboratory abnormalities (peak values of uric acid, potassium, phosphorus, calcium, LDH, and creatinine), management strategies (hydration, urate-lowering agents, dialysis), and clinical outcomes (survival or death).

### 2.5 Risk of bias assessment

Methodological quality of included case reports was assessed using the Joanna Briggs Institute (JBI) Critical Appraisal Checklist for Case Reports (19). The checklist evaluates eight domains: demographics, patient history, clinical presentation, diagnostic tests, intervention, post-intervention course, adverse events, and takeaway lessons. Each item was rated as “yes, “ “no, “ “unclear, “ or “not applicable.” The JBI assessments were performed independently by two reviewers (L.H.Z. and Y.C.), with disagreements resolved through discussion with a third reviewer (M.Z.).

### 2.6 Data synthesis

Due to the inherent heterogeneity of case reports and the absence of comparison groups, no meta-analysis was performed. Data were summarized descriptively using frequencies, proportions, and ranges. Results were stratified by precipitating factors where sample size permitted. All analyses were narrative and presented in tabular format.

## 3 Results

### 3.1 Literature search and study selection

The systematic search of PubMed, Embase, and Web of Science yielded a total of 597 records. An additional 13 records were identified through handsearching of reference lists and targeted journal searches. After removing 123 duplicates, 474 records remained for title and abstract screening. Of these, 442 records were excluded based on irrelevance to the study question (non-TLS, non-breast cancer, non-case reports, non-English, conference abstracts, or basic research). The remaining 32 records underwent full-text assessment, of which 11 were excluded: 4 for failure to meet Cairo-Bishop TLS diagnostic criteria, 3 for lack of individual patient-level data, and 4 for being conference abstracts, reviews, or commentaries without original case data. A total of 20 publications were ultimately included (**Table 1**). Among these, two publications reported 2 cases each (Mott 2005; Nishimura 2015) (21, 22), resulting in 21 individual cases. One case Omori et al. (20) described successful TLS prevention and was excluded from the primary analysis; the remaining 20 cases developed TLS and were included in the mortality calculations. The PRISMA flow diagram is presented in **Figure 1**.

**Table 1:** Summary of included case reports of tumor lysis syndrome in breast cancer (2000–2026).

First author	Year	Age/Sex	Subtype	Metastatic sites	Precipitant	Time to TLS	Outcome
Endocrine therapy(n=4)							
Kikuchi M	2024	80/F	IDC, ER+/PR+/HER2 1+	Local advanced (chest wall)	Letrozole	7 days	Died
Zigrossi P	2001	76/F	IDC, ER+/PR+	Pleura, pericardium	Letrozole	2 days	Survived
Watkinson GE	2021	74/F	Occult adenocarcinoma, ER+/HER2-	Pleura, mediastinal LNs	Letrozole	4 days	Died
Tasnim N	2026	67/F	Adenocarcinoma, ER+	Chest wall, thoracic LNs, pleura	Anastrozole	<48 h	Died
HER2-targeted therapy(n=4)							
Taira F	2015	69/F	IDC, triple-positive	Liver	Trastuzumab	6 days	Died
Baudon C	2016	58/F	IDC, ER-/PR-/HER2+	Liver, lung, chest wall	Trastuzumab + pertuzumab	1 day	Died
Nishimura K (1)	2015	46/F	HER2+ metastatic	Multiple organs	Lapatinib + capecitabine	6 days	Survived
Nishimura K (2)	2015	66/F	HER2+ metastatic	Multiple organs	Lapatinib	7 days	Survived
Chemotherapy(n=6)							
Aslam HM	2019	58/F	IDC, ER-/PR-/HER2+	Liver, lung, bone	Gemcitabine	4 days	Died
Kawaguchi Ushio A	2013	59/F	Adenocarcinoma	Bone	Gemcitabine	16 days	Survived
Vaidya GN	2015	52/F	IDC, ER+/PR+/HER2-	Liver, bone	Paclitaxel	7 days	Died
Mott FE (1)	2005	47/F	IDC, ER+/PR-/HER2-	Lung, bone, kidney	FEC (5-FU/epirubicin/C)	1 day	Survived
Mott FE (2)	2005	44/F	IDC, ER+/PR+/HER2+	Liver, bone	Gemcitabine + cisplatin	1 day	Survived
Kurt M	2004	42/F	IDC (stage IIB)	Liver	Capecitabine	<12 h	Died
Radiotherapy(n=2)							
Furusawa M	2023	65/F	IDC, ER+/PR+/HER2-	Local, lung, bone	Palliative RT (30 Gy/10fr)	<12 h	Survived
Rostom AY	2000	73/M	IDC, grade 3	Liver, lung, bone	Hemi-body RT (8.5 Gy)	2 days	Died
Novel targeted/immunotherapy(n=2)							
Handy C	2021	66/F	HR+/HER2-, PIK3CA+	Liver, bone, brain, peritoneum	Alpelisib + fulvestrant	12 days	Survived
Carrier X	2020	55/F	mTNBC (EGFR amp, PIK3CA+)	Liver, bone	Atezolizumab + nab-paclitaxel	6 days	Survived
Spontaneous(n=2)							
Emadzadeh A	2025	62/F	Metastatic breast cancer	Liver	Spontaneous	—	Died
Parsi M	2019	36/F	IDC, ER+/PR+/HER2+	Lung, liver, bone, LNs	Spontaneous	—	Survived
Prevention case (n=1)							
Omori S*	2023	41/F	IDC, HR+/HER2+	Liver, bone, lung	Trastuzumab + pertuzumab (prophylaxis)	—	Prevented*

F, female; M, male; IDC, invasive ductal carcinoma; ER, estrogen receptor; PR, progesterone receptor; HER2, human epidermal growth factor receptor 2; HR, hormone receptor; LNs, lymph nodes; RT, radiotherapy; FEC, 5-fluorouracil/epirubicin/cyclophosphamide; mTNBC, metastatic triple-negative breast cancer; amp, amplification; h, hours.

*Omori et al. (20): prevention case (TLS did not occur), excluded from primary analysis and mortality calculation. Mott 2005 and Nishimura 2015 each reported 2 cases.

**Figure 1:**
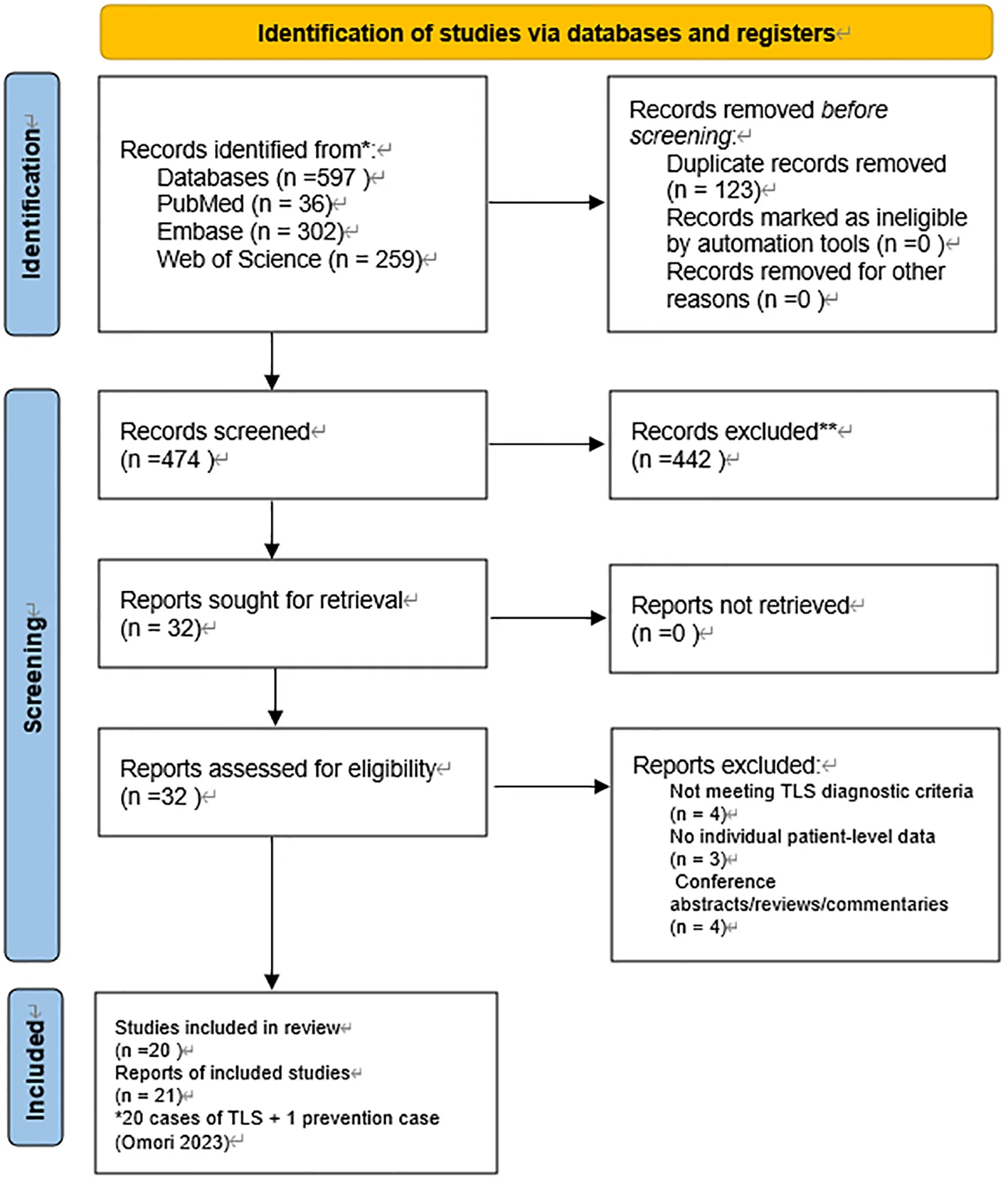
PRISMA 2020 flow diagram of study selection for systematic review of tumor lysis syndrome in breast cancer.

### 3.2 Study and patient characteristics

A total of 21 cases were included in the summary (20 TLS cases and 1 prevention case), published between 2000 and 2026, with a notable clustering of cases after 2019 (n=14, 66.7%), reflecting increased reporting in recent years. All cases were female except one male patient (17). Patient age ranged from 36 to 80 years, with a median age of 59.5 years (IQR: 47.0–67.0).

All patients had stage IIB–IV disease, predominantly stage IV (90%, 18/20), with 2 stage IIIB cases (Kikuchi 2024; Kurt 2004) and 1 stage IIB case (Taira 2015).The most common breast cancer subtype was hormone receptor-positive/HER2-negative (n=8, 36.4%), followed by HER2-positive (n=7, 31.8%), and triple-negative (n=3, 13.6%). Subtype was not reported in 4 cases (18.2%).

Metastatic involvement was documented in 19 of 20 TLS cases (95%), with one locally advanced case without distant metastasis (Kikuchi 2024) (13). The most frequently involved sites were liver (n=11, 55%), followed by bone (n=10, 50%), lung (n=5, 25%), and pleura/mediastinal/chest wall (n=4, 20%). An additional two cases (Nishimura 2015) (22) were reported as having multi-organ metastases without specific site details. Notably, all patients with liver metastases had either stage IV disease or locally advanced disease with extensive hepatic involvement (11). Detailed characteristics of each included case are summarized in **Table 1**. The methodological quality of the included case reports was assessed using the JBI Critical Appraisal Checklist for Case Reports. All included studies met at least 6 of the 8 criteria, with the most common limitation being incomplete reporting of patient history and adverse events. Detailed JBI quality assessments for each included study are provided in **Supplementary Table S2**.

### 3.3 Precipitating factors and stratified outcomes

The precipitating factors of TLS were categorized into six groups: endocrine therapy (n=4), HER2-targeted therapy (n=4), chemotherapy (n=6), radiotherapy (n=2), novel targeted/immunotherapy (n=2), and spontaneous TLS (n=2). The overall mortality rate was 50% (10/20) among patients who developed TLS. The distribution of cases, mortality rates, and time to onset for each category are summarized in **Table 2**.

**Table 2:** Clinical outcomes of tumor lysis syndrome in breast cancer stratified by precipitating factors.

Precipitant category	Cases, n	Survived, n	Died, n	Mortality rate	Median time to onset (range)
Endocrine therapy	4	1	3	75%	4 days (<48 h-7 days)
HER2-targeted therapy	4	2	2	50%	6 days (1–7 days)
Chemotherapy	6	3	3	50%	4 days (<12 h-16 days)
Radiotherapy	2	1	1	50%	1.5 days (<12 h-2 days)
Novel targeted/immunotherapy	2	2	0	0%	9 days (6–12 days)
Spontaneous	2	1	1	50%	—
Total	20	10	10	50%	—

*Omori et al. (20): prevention case (TLS did not occur), excluded from the primary analysis and mortality calculation.

#### 3.3.1 Endocrine therapy

Four patients developed TLS following treatment with aromatase inhibitors, including letrozole (n=3) and anastrozole (n=1). This group had the highest mortality rate among all categories (75%, 3/4), with time to onset ranging from 2 to 7 days. Kikuchi et al. (13) reported an 80-year-old patient with locally advanced breast cancer who developed TLS on day 7 after letrozole initiation; despite hydration, febuxostat, and hemodialysis, the patient died on day 4 of hospitalization. Watkinson et al. (23) described a 74-year-old patient with occult breast cancer who was diagnosed with TLS on day 4 after letrozole treatment and died in hospice care. Tasnim et al. (14) reported a 67-year-old patient with ER-positive advanced breast cancer who developed TLS within 48 hours of anastrozole initiation (14), complicated by atrial fibrillation, respiratory failure, and acute ischemic stroke; the patient died of cardiac arrest despite aggressive supportive care. The only survivor in this group was reported by Zigrossi et al. (24): a 76-year-old patient with advanced breast cancer who presented with shock, bilateral pleural effusion, and cardiac tamponade after letrozole treatment (24), gradually recovered through supportive care, and maintained complete remission for 20 months.

#### 3.3.2 HER2-targeted therapy

Four patients developed TLS during HER2-targeted therapy, with a mortality rate of 50% (2/4). Taira et al. (25) reported a 69-year-old patient with triple-positive breast cancer and multiple liver metastases who developed TLS on day 6 after trastuzumab treatment, presenting with arrhythmia, hyperkalemia (8.1 mmol/L), and hyperuricemia (22.2 mg/dL); the patient died on day 11 due to the contraindication of hemodialysis because of severe hepatic dysfunction. Baudon et al. (26) described a 58-year-old patient with HER2-positive breast cancer and liver/lung metastases who developed hypovolemic shock and multiple organ failure on day 2 after a single dose of trastuzumab and pertuzumab, and died despite optimal management. Two patients treated with lapatinib-based regimens both survived (Nishimura et al (22).), with time to onset of 6 and 7 days, respectively.

#### 3.3.3 Chemotherapy

Chemotherapy was the most common precipitant (n=6), with a mortality rate of 50% (3/6). The agents involved included gemcitabine (n=3), paclitaxel (n=1), capecitabine (n=1), and the FEC regimen (n=1). Aslam et al. (27) reported a 58-year-old patient with HER2-positive (ER−/PR−) disease who developed TLS on day 4 after a single dose of gemcitabine and was subsequently transferred to hospice care (27). Vaidya et al. (28) described a 52-year-old patient with HR+/HER2- disease who developed TLS on day 7 after paclitaxel and died during hospitalization (28). Kurt et al. (29) reported a 42-year-old patient who developed TLS 11 hours after capecitabine and died shortly thereafter (29). Among the three survivors, Kawaguchi Ushio et al. (18) reported a 59-year-old patient with bone metastases who developed TLS on day 16 after gemcitabine, which was successfully managed with rasburicase. Mott et al. (21) reported two patients who developed TLS on day 1 after receiving FEC and gemcitabine plus cisplatin, respectively; both recovered with hydration, allopurinol, and rasburicase (21).

#### 3.3.4 Radiotherapy

Two patients developed TLS following radiotherapy, with one death and one survival. Rostom et al. (17) reported a 73-year-old male patient with breast cancer who developed TLS 48 hours after hemi-body irradiation (8.5 Gy); despite allopurinol and hydration, the patient progressed to coma and died on day 8 (17). Furusawa et al. (30) described a 65-year-old patient with HR+/HER2- disease who developed TLS 11 hours after palliative radiotherapy (30 Gy/10 fractions) to cervical metastatic lesions; the patient recovered with hemodialysis and electrolyte correction (30), representing a rare survival case in radiotherapy-associated TLS.

#### 3.3.5 Novel targeted/immunotherapy

Two patients developed TLS following novel therapeutic agents, and both survived. Handy et al. (11) reported a 66-year-old patient with HR+/HER2-, PIK3CA-mutated metastatic breast cancer who developed TLS on day 12 after initiating alpelisib plus fulvestrant (11), presenting with creatinine 15.15 mg/dL, potassium 8.2 mmol/L, and uric acid 16.2 mg/dL; renal function recovered to baseline with aggressive hydration, allopurinol, and rasburicase, without requiring renal replacement therapy. Carrier et al. (12) reported a 55-year-old patient with metastatic triple-negative breast cancer who developed TLS on day 6 after atezolizumab plus nab-paclitaxel (12); symptoms completely resolved within 4 days after a single dose of rasburicase. These represent the first reported TLS cases associated with a PI3K inhibitor and an immune checkpoint inhibitor in breast cancer, respectively.

#### 3.3.6 Spontaneous TLS

Two patients developed spontaneous TLS without receiving any anticancer therapy, with one death and one survival. Emadzadeh et al. (31) reported a 62-year-old patient with liver metastases who presented with TLS and died of refractory metabolic acidosis (31). Parsi et al. (32) described a 36-year-old patient with ER+/PR+/HER2+ extensive metastatic disease who met TLS diagnostic criteria on admission and recovered with aggressive hydration and rasburicase.

In addition to the six precipitant categories described above, one case of successful TLS prevention was identified. Omori et al. (20) reported a 41-year-old patient with HER2-positive, hormone receptor-positive breast cancer and multiple liver and bone metastases who received prophylactic hydration and febuxostat before initiating trastuzumab and pertuzumab. TLS did not occur, though the patient developed disseminated intravascular coagulation on day 1 of treatment and subsequently received reduced-dose paclitaxel without further complications. This case is summarized in **Table 1** but was excluded from the primary analysis and mortality calculations.

### 3.4 Clinical presentation, management, and outcomes

Among the 20 patients who developed TLS, acute kidney injury (AKI) was the most common clinical manifestation, observed in all cases. The severity of AKI ranged from mild elevation of serum creatinine to cases requiring renal replacement therapy. Cardiac involvement was also frequently observed, including arrhythmias (14, 25), electrocardiographic abnormalities (11), hypovolemic shock (26), and cardiac tamponade (24). Respiratory system involvement mainly manifested as respiratory failure (14) or acute respiratory distress syndrome (13). Some patients presented with altered mental status (23, 28), and one case was complicated by disseminated intravascular coagulation (20).

All cases exhibited elevated uric acid and lactate dehydrogenase (LDH) levels, indicating massive tumor cell breakdown. Peak uric acid levels ranged from 11.4 mg/dL to 22.2 mg/dL; peak potassium levels ranged from 6.0 mmol/L to 8.2 mmol/L; the highest recorded peak phosphorus level was 13.0 mg/dL; hypocalcemia was observed in a subset of cases. In the anastrozole-associated TLS case reported by Tasnim et al. (14), LDH surged from a baseline of 188 U/L to 3, 052 U/L, with a phosphorus level of 13.0 mg/dL, reflecting extremely rapid and extensive tumor lysis.

All patients received intravenous hydration. For urate-lowering therapy, 13 patients received allopurinol or febuxostat, and 9 received rasburicase; 8 patients required hemodialysis due to severe AKI or refractory electrolyte disturbances. In some cases, anticancer therapy was temporarily discontinued upon TLS diagnosis and was subsequently resumed at reduced doses after clinical stabilization (11, 20). Zigrossi et al. (24) reported a case of letrozole-associated TLS in which the patient survived a 2-week critical period after drug discontinuation and supportive care.

Among the 20 patients who developed TLS, 10 died, yielding an overall mortality rate of 50%. Most deaths occurred within days after TLS diagnosis, with the primary causes being multiple organ failure (26), cardiac arrest (14), and refractory metabolic acidosis (31). Of the 9 patients who received rasburicase, 6 survived (66.7%), whereas only 4 of the 13 patients who received allopurinol or febuxostat survived (30.8%). Although statistical comparison was not feasible due to the small sample size, this descriptive observation does not allow causal inference regarding treatment-specific effects.Outcomes stratified by precipitant category are detailed in **Table 2**. Detailed patient-level laboratory and management data, including baseline renal function where available, are provided in **Supplementary Table S3**.

### 3.5 Novel therapeutic agents and special cases

Two cases of TLS were associated with novel therapeutic agents. Handy et al. (11) reported a 66-year-old patient with HR+/HER2−, PIK3CA-mutated metastatic breast cancer who developed TLS 12 days after initiating alpelisib (a PI3K inhibitor) combined with fulvestrant. Laboratory findings revealed creatinine 15.15 mg/dL, potassium 8.2 mmol/L, and uric acid 16.2 mg/dL. The patient recovered with hydration, allopurinol, and rasburicase, with renal function returning to baseline without requiring renal replacement therapy. Alpelisib was subsequently resumed at a reduced dose without TLS recurrence. Carrier et al. (12) reported a 55-year-old patient with metastatic triple-negative breast cancer who developed TLS 6 days after receiving atezolizumab (a PD-L1 inhibitor) plus nab-paclitaxel. Symptoms completely resolved within 4 days after a single dose of rasburicase. These two cases represent the first reported TLS cases associated with a PI3K inhibitor and an immune checkpoint inhibitor in breast cancer, respectively.

One additional case deserves special mention regarding TLS prevention. Omori et al. (20) reported a 41-year-old patient with HER2-positive/HR-positive breast cancer and multiple liver and bone metastases, presenting with high-risk features including elevated LDH (4, 361 U/L) and hyperuricemia (9.8 mg/dL). Before initiating trastuzumab plus pertuzumab, the patient received prophylactic hydration and febuxostat. Although disseminated intravascular coagulation (DIC) developed on day 1 of treatment, TLS was successfully prevented. The patient subsequently received reduced-dose paclitaxel without life-threatening complications and achieved a partial response after four cycles of anti-HER2 therapy and chemotherapy. This case suggests that prophylactic measures may be effective in reducing TLS risk in breast cancer patients with high tumor burden and elevated baseline LDH and uric acid levels.

## 4 Discussion

This systematic review summarizes 20 cases of TLS in breast cancer reported between 2000 and 2026, with one additional case of successful TLS prevention, representing the largest literature synthesis in this field to date. Several observations merit discussion.

First, TLS in breast cancer is not as rare as traditionally believed. Among the 21 cases, 14 (66.7%) were published after 2019, accounting for nearly two-thirds of all cases. This increasing trend may reflect improved clinical recognition and more standardized case reporting in recent years, but the widespread use of novel anti-tumor agents may also have contributed to a true increase in TLS incidence. This finding challenges the conventional view that breast cancer is a low-risk malignancy for TLS and calls for updated clinical awareness.

Second, the spectrum of precipitating factors for TLS has broadened considerably beyond conventional chemotherapy. In this case series, TLS was triggered by endocrine therapy (n=4), HER2-targeted therapy (n=4), radiotherapy (n=2), PI3K inhibitors (n=1), and immunotherapy (n=1), in addition to chemotherapy (n=6). Among these, endocrine therapy-associated TLS carried the highest mortality rate (75%, 3/4), a finding that warrants particular attention in clinical practice.

Third, the overall prognosis of breast cancer patients who develop TLS remains poor. The mortality rate in this cohort was 50% (10/20), comparable to that reported in systematic reviews of TLS in colorectal cancer (59%) and lung cancer (48%), but considerably higher than the in-hospital mortality rate of approximately 21% reported for TLS in hematologic malignancies. This observation supports the notion that TLS in solid tumors carries a more fulminant course and a worse prognosis than in hematologic cancers, emphasizing the need for prompt recognition and aggressive intervention.

### 4.1 Endocrine therapy-associated TLS

In this series, 4 cases of TLS were associated with endocrine therapy, accounting for 19.0% of all cases (4/21), but the mortality rate reached 75% (3/4), the highest observed among all precipitant categories. All 4 cases were related to aromatase inhibitors, including letrozole (n=3) and anastrozole (n=1), suggesting that TLS can occur in temporal association with these agents under specific clinical conditions, and they should not be regarded as entirely without risk of acute metabolic complications.

All 4 patients had hormone receptor-positive advanced breast cancer, with a concentrated high-risk profile characterized by advanced age and high tumor burden. Patient ages ranged from 67 to 80 years, all being elderly; 3 had distant metastases involving the pleura or liver, and only 1 had locally advanced disease (Kikuchi et al. (13)). Regarding the timing of onset, endocrine therapy-associated TLS occurred predominantly between 2 and 7 days after drug initiation, with a relatively early and concentrated time frame. The anastrozole-associated case reported by Tasnim et al. (14) developed typical TLS manifestations within 48 hours of drug administration. This clinical pattern challenges the traditional view that “endocrine therapy acts slowly and does not induce massive tumor lysis in the short term, “ indicating that for advanced breast cancer patients with high tumor burden, aromatase inhibitors can still trigger large-scale tumor necrosis and acute TLS in a short period.

The outcomes of endocrine therapy-associated TLS in this series showed a clear dichotomy. The only survivor was the letrozole-associated case reported by Zigrossi et al. (24), who presented with a critical condition complicated by shock, bilateral pleural effusion, and cardiac tamponade. After nearly two weeks of intensive care, the patient survived the acute phase and achieved a 20-month complete remission with significant long-term survival benefit. The remaining 3 patients died despite active treatment, with the main causes being multiple organ failure, acute respiratory distress syndrome (ARDS), and cardiac arrest—all severe systemic complications of TLS.

The high mortality of endocrine therapy-associated TLS is not attributable to TLS itself alone, but rather to the combined effect of treatment-related factors and baseline high-risk patient characteristics. The patients in this group were generally elderly, had poor baseline functional status, multiple distant metastases, and high tumor burden, all of which contributed to the severity of TLS and ultimately led to poor outcomes. Current TLS risk assessment systems are primarily designed for chemotherapy and hematologic malignancies and have not adequately incorporated the risk weight of endocrine therapy, resulting in long-term underestimation of TLS risk associated with aromatase inhibitors. Given the widespread use of aromatase inhibitors in hormone receptor-positive breast cancer, clinicians should carefully assess patient age, tumor burden, renal function, and baseline electrolyte and metabolic status before initiating such therapy. For high-risk patients—particularly those who are elderly or have high metastatic burden—enhanced dynamic monitoring during the early phase of treatment is essential to enable early identification of TLS prodromes and timely intervention, thereby reducing the risk of severe complications and mortality.

### 4.2 HER2-targeted therapy and novel anticancer agent-associated TLS

In this series, 4 cases of TLS were associated with HER2-targeted therapy, accounting for 19.0% of all cases (4/21), with a mortality rate of 50% (2/4). All 4 patients had HER2-positive advanced breast cancer; 2 received trastuzumab or trastuzumab plus pertuzumab, and 2 received lapatinib. The time to onset ranged from 1 to 7 days after treatment initiation. In the case reported by Baudon et al. (26), TLS developed on day 2 after the first dose of trastuzumab plus pertuzumab, presenting with hypovolemic shock and multiple organ failure. Notably, in the trastuzumab-associated TLS case reported by Taira et al. (25), the patient died because severe hepatic dysfunction precluded hemodialysis, highlighting that baseline liver and kidney function directly affect the feasibility of salvage interventions once TLS occurs.

Both lapatinib-associated TLS cases survived. Whether this reflects a more favorable safety profile of lapatinib as an oral targeted agent with relatively milder pharmacodynamics, or simply earlier recognition and intervention before irreversible organ damage occurred, cannot be determined from the available data. Nonetheless, TLS risk associated with lapatinib in heavily pretreated HER2-positive advanced breast cancer also warrants clinical attention.

Two additional cases were associated with novel anticancer agents. Handy et al. (11) reported a case of TLS occurring on day 12 after treatment with alpelisib (a PI3K inhibitor) plus fulvestrant, presenting with extreme laboratory abnormalities: creatinine 15.15 mg/dL, potassium 8.2 mmol/L, and uric acid 16.2 mg/dL. Renal function recovered completely after hydration, allopurinol, and rasburicase, and alpelisib was subsequently resumed at a reduced dose without TLS recurrence. Carrier et al. (12) reported a case of TLS occurring on day 6 after treatment with atezolizumab (a PD-L1 inhibitor) plus nab-paclitaxel, with complete resolution within 4 days after a single dose of rasburicase. These two cases represent the first reported TLS cases associated with a PI3K inhibitor and an immune checkpoint inhibitor in breast cancer, respectively. Their shared feature is that both involve novel mechanisms of action with rapid antitumor activity that may precipitate extensive tumor cell breakdown, but timely recognition and aggressive management led to favorable outcomes.

Across the 6 cases of HER2-targeted therapy and novel anticancer agent-associated TLS combined, the overall mortality rate was 33.3% (2/6), lower than that of the endocrine therapy and chemotherapy groups. However, the deceased patients in these groups shared a common feature: severe baseline liver metastases or hepatic dysfunction, consistent with the well-established literature suggesting liver metastasis as a potential risk factor for TLS. For patients with advanced breast cancer receiving HER2-targeted therapy or novel anticancer agents, particularly those with liver metastases or baseline hepatic/renal impairment, close TLS monitoring during the early treatment phase remains essential.

### 4.3 Chemotherapy-associated TLS

In this series, 6 cases of TLS were associated with chemotherapy, accounting for 28.6% of all cases (6/21), with a mortality rate of 50% (3/6). The chemotherapeutic agents involved were all commonly used in breast cancer, including gemcitabine (n=3), paclitaxel (n=1), capecitabine (n=1), and the FEC regimen (n=1). The predominance of gemcitabine-related cases (3 of 6) and the presence of cases across multiple drug classes suggest that chemotherapy-associated TLS is not agent-specific. Patient-related factors—including tumor burden, chemosensitivity, and baseline performance status—may play a more critical role than the choice of cytotoxic drug.

The time from chemotherapy initiation to TLS onset varied considerably across cases. The shortest interval was 11 hours (Kurt 2004, capecitabine), while the longest was 16 days (Kawaguchi Ushio 2013, gemcitabine). This wide variation challenges the conventional practice of limiting TLS monitoring to the first 24–72 hours after chemotherapy administration. For patients with high-risk features, extended monitoring beyond the traditional window may be necessary to avoid missed or delayed diagnoses.

All 3 fatal cases had liver metastases, with 2 having extensive hepatic involvement (27, 28) and 1 presenting with ultra-rapid onset (29). These observations suggest that patients with liver metastases and high tumor burden are particularly difficult to salvage once TLS develops, likely due to impaired metabolic clearance and reduced renal reserve. In contrast, all 3 surviving cases benefited from timely intervention: Kawaguchi Ushio et al. (18) improved with rasburicase monotherapy, and the 2 cases reported by Mott et al. (21) recovered fully with hydration combined with allopurinol and rasburicase. These outcomes underscore the potential benefit of early rasburicase use in chemotherapy-associated TLS.

Although recent years have seen increasing reports of TLS associated with novel targeted and endocrine therapies, chemotherapy remains the most frequently reported precipitant in breast cancer-associated TLS. The clinical relevance of chemotherapy-related TLS should not be overlooked, and clinicians should maintain vigilance for TLS risk assessment, early monitoring, and prompt intervention, even as attention shifts toward emerging therapeutic modalities.

### 4.4 Radiotherapy and spontaneous TLS

Two cases of radiotherapy-associated TLS were identified. Rostom et al. (17) reported a 73-year-old male patient who developed TLS 48 hours after hemi-body irradiation (8.5 Gy); despite allopurinol and hydration, the patient progressed to coma and died on day 8. Furusawa et al. (30) reported a 65-year-old patient with HR+/HER2− breast cancer who received palliative radiotherapy (30 Gy/10 fractions) for cervical lymph node metastases and developed TLS 11 hours after the first fraction; the patient recovered with hemodialysis and electrolyte correction. Both patients had extensive metastatic disease, suggesting that even localized palliative radiotherapy can precipitate TLS in patients with high tumor burden. Although radiotherapy-associated TLS is rare, it is not nonexistent, and patients with advanced breast cancer receiving radiotherapy should be included in TLS monitoring protocols.

Two cases of spontaneous TLS were identified, with one death and one survival. Emadzadeh et al. (31) reported a 62-year-old patient with liver metastases who presented with TLS without prior anticancer therapy and died of refractory metabolic acidosis. Parsi et al. (32) reported a 36-year-old patient with ER+/PR+/HER2+ extensive metastatic disease who met TLS criteria on admission and recovered with aggressive hydration and rasburicase. Although spontaneous TLS is uncommon, it can occur in patients with newly diagnosed advanced breast cancer. For patients presenting with liver metastases, high tumor burden, hyperuricemia, or markedly elevated LDH, TLS risk should be assessed even before anticancer therapy is initiated.

### 4.5 Clinical implications and risk assessment

The findings of this review have several implications for clinical practice.Current TLS risk assessment systems are primarily derived from studies on hematologic malignancies and cytotoxic chemotherapy, with solid tumors historically classified as low-risk. However, 14 of 21 cases (66.7%) in this series were published after 2019, and precipitants included endocrine therapy, HER2-targeted therapy, immunotherapy, and other novel agents, indicating that the traditional risk stratification framework no longer fully reflects the contemporary therapeutic landscape in breast cancer. Continuing to uniformly classify breast cancer as “low-risk” may lead to insufficient clinical vigilance and delayed diagnosis and intervention.

Liver metastasis emerged as the most common metastatic site (55%, 11/20) in this series, and the majority of fatal cases had liver involvement, consistent with previous literature identifying liver metastasis as a commonly observed feature among the reported cases. Hepatic metastases may increase TLS risk through multiple mechanisms, including increased total tumor burden, impaired uric acid metabolism, and reduced renal functional reserve. For breast cancer patients with liver metastases, regardless of the treatment modality, they should be considered at high risk for TLS, with baseline assessment before treatment and dynamic monitoring of metabolic parameters and organ function throughout therapy.

Regarding management, 6 of 9 patients receiving rasburicase survived (66.7%), compared with 4 of 13 patients receiving allopurinol or febuxostat (30.8%). Although statistical comparison was not feasible due to the small sample size, this descriptive difference may warrant attention. Rasburicase has a rapid onset of action and potent urate-lowering effects, making it more suitable for high-risk patients or those with established TLS. Current indications for rasburicase in solid tumor TLS remain limited, with use largely restricted to hematologic malignancies in most centers. The data from this review support more proactive consideration of rasburicase in breast cancer-associated TLS.

Regarding prevention, only one case (Omori et al. (20)) received targeted prophylaxis (hydration plus febuxostat) before treatment and successfully avoided TLS. This patient had clearly identifiable high-risk features (multiple liver metastases, LDH 4, 361 U/L, uric acid 9.8 mg/dL), suggesting that preventive measures can be effective in appropriately selected high-risk patients. The current challenge lies in the absence of validated risk stratification tools specifically for breast cancer patients. Future research should focus on developing TLS risk assessment models for solid tumors, incorporating tumor burden, metastatic sites (particularly liver), baseline LDH and uric acid levels, and treatment modality into a comprehensive evaluation to guide individualized prevention strategies.

### 4.6 Limitations

This review has several limitations. First, all included studies were case reports or case series, representing the lowest level of evidence in clinical research. The absence of control groups precludes calculation of TLS incidence or establishment of causal relationships between specific treatments and TLS. Second, publication bias is inherent to case report-based reviews. Severe or fatal cases are more likely to be reported, whereas mild cases or those with successful prevention may remain unpublished, potentially leading to overestimation of mortality rates and severity of breast cancer-associated TLS. Third, data reporting across included cases was inconsistent; some reports lacked detailed laboratory values, treatment protocols, or long-term follow-up information, limiting the depth of subgroup analyses. Fourth, this review predominantly included English-language publications. One Japanese-language article was included based on the clinical data available in its English abstract, with data extraction limited to the information available therein, which may have introduced reporting gaps. Finally, heterogeneity in TLS diagnostic criteria, treatment approaches, and outcome definitions across studies is unavoidable in case report-based systematic reviews. Despite these limitations, this review represents the largest literature synthesis on TLS in breast cancer to date and provides valuable reference data for clinical practice.

## 5 Conclusions

This systematic review summarizes 20 cases of TLS in breast cancer reported between 2000 and 2026, with one additional case of successful TLS prevention, representing the largest literature synthesis in this field to date. The findings indicate that TLS in breast cancer is not as rare as traditionally believed, with nearly two-thirds of cases published after 2019. The spectrum of precipitating factors has expanded beyond conventional chemotherapy to include endocrine therapy, HER2-targeted therapy, immunotherapy, and PI3K inhibitors. The overall mortality rate was 50% (10/20), comparable to that reported in other solid tumors but higher than in hematologic malignancies.

Endocrine therapy-associated TLS carried the highest mortality rate (75%), with aromatase inhibitors (letrozole and anastrozole) as the primary triggers, challenging the traditional view that endocrine therapy is slow-acting and unlikely to induce TLS. Liver metastasis was the most common high-risk factor (55%, 11/20), and the majority of fatal cases had liver involvement. The survival rate in patients receiving rasburicase (66.7%) was higher than in those receiving allopurinol or febuxostat (30.8%), suggesting that rasburicase may offer a survival benefit in breast cancer TLS management, although statistical comparison was not feasible due to the small sample size.

Current TLS risk assessment systems, primarily developed for chemotherapy and hematologic malignancies, are no longer fully applicable to the contemporary treatment landscape of breast cancer. There is an urgent need to develop breast cancer-specific TLS risk stratification tools that incorporate tumor burden, liver metastasis, baseline LDH and uric acid levels, and treatment modality to enable individualized risk assessment and targeted prevention strategies.

## Data Availability

The original contributions presented in the study are included in the article/**Supplementary Material**. Further inquiries can be directed to the corresponding author.
