# Supplementary material for: Tumor lysis syndrome in breast cancer: a systematic review of the literature
Source: Front Oncol. 2026 Oct 14;16:1937565. doi: 10.3389/fonc.2026.1937565 (PMC13713353; doi:10.3389/fonc.2026.1937565)
Supplement: Supplementary file 1 [file Table1.docx]

**Table S1. Search Strategies**

| **Databases** | **Search** | **Query** | **No. of Results** |
| --- | --- | --- | --- |
| **PubMed**  Search Date: June 25, 2026  Final Search: #8 | #1 | " Tumor Lysis Syndrome"[Mesh] | 4,862 |
|  | #2 | "tumor lysis syndrome"[Title/Abstract] OR TLS[Title/Abstract] | 3,214 |
|  | #3 | #1 OR #2 | 5,891 |
|  | #4 | "Breast Neoplasms"[MeSH] | 384,505 |
|  | #5 | "breast cancer"[Title/Abstract] OR "breast carcinoma"[Title/Abstract] OR "mammary cancer"[Title/Abstract] | 427,481 |
|  | #6 | #4 OR #5 | 691,26 |
|  | #7 | #3 AND #6 | 123 |
|  | #8 | #7 AND English[Language] AND (Case Reports[pt] OR Review[pt]) AND 2000:2026[dp] | 36 |
| **Embase**  Search Date: June 25, 2026 | #1 | 'tumor lysis syndrome'/exp | 9,874 |
|  | #2 | 'tumor lysis syndrome':ab,ti OR tls:ab,ti | 5,642 |
|  | #3 | #1 OR #2 | 11,208 |
|  | #4 | 'breast cancer'/exp | 717,188 |
|  | #5 | 'breast cancer':ab,ti OR 'breast carcinoma':ab,ti | 640,621 |
|  | #6 | #4 OR #5 | 21,979 |
|  | #7 | #3 AND #6 | 578 |
|  | #8 | #7 AND [2000-2026]/py AND [english]/lim AND ([article]/lim OR [review]/lim) | 302 |
| **Web of Science**  Search Date: June 25, 2026  Final Search: #4 | #1 | TS=("tumor lysis syndrome" OR TLS) | 5,234 |
|  | #2 | TS=("breast cancer" OR "breast carcinoma" OR "mammary cancer") | 596,789 |
|  | #3 | #1 AND #2 | 387 |
|  | #4 | #3 AND PY=(2000-2026) AND (DT=Article OR DT=Review) AND LA=English | 259 |
