# Supplementary material for: Tumor lysis syndrome in breast cancer: a systematic review of the literature
Source: Front Oncol. 2026 Oct 14;16:1937565. doi: 10.3389/fonc.2026.1937565 (PMC13713353; doi:10.3389/fonc.2026.1937565)
Supplement: Supplementary file 2 [file Table2.docx]

**Supplementary Table S2. JBI Critical Appraisal Checklist for Case Reports – Individual Study Quality Assessment**

| First author | Year | Demographic characteristics | Medical history | Clinical presentation | Diagnostic workup | Intervention details | Post-intervention course | Adverse events | Take-home lessons | Total (/8) |
| --- | --- | --- | --- | --- | --- | --- | --- | --- | --- | --- |
| Kikuchi M | 2024 | Yes | Yes | Yes | Yes | Yes | Yes | Yes | Yes | 8 |
| Zigrossi P | 2001 | Unclear | Unclear | Yes | Yes | Yes | Yes | Yes | Yes | 6 |
| Watkinson GE | 2021 | Yes | Yes | Yes | Yes | Yes | Yes | Yes | Yes | 8 |
| Tasnim N | 2026 | Yes | Yes | Yes | Yes | Yes | Yes | Yes | Yes | 8 |
| Taira F | 2015 | Yes | Yes | Yes | Yes | Yes | Yes | Yes | Yes | 8 |
| Baudon C | 2016 | Yes | Yes | Yes | Yes | Yes | Yes | Yes | Yes | 8 |
| Nishimura K (1) | 2015 | Unclear | Unclear | Unclear | Unclear | Yes | Yes | Unclear | Unclear | 2 |
| Nishimura K (2) | 2015 | Unclear | Unclear | Unclear | Unclear | Yes | Yes | Unclear | Unclear | 2 |
| Aslam HM | 2019 | Yes | Yes | Yes | Yes | Yes | Yes | Yes | Yes | 8 |
| Kawaguchi Ushio A | 2013 | Unclear | Unclear | Yes | Yes | Yes | Yes | Yes | Unclear | 5 |
| Vaidya GN | 2015 | Yes | Yes | Yes | Yes | Yes | Yes | Yes | Yes | 8 |
| Mott FE (1) | 2005 | Yes | Yes | Yes | Yes | Yes | Yes | Yes | Yes | 8 |
| Mott FE (2) | 2005 | Yes | Yes | Yes | Yes | Yes | Yes | Yes | Yes | 8 |
| Kurt M | 2004 | Unclear | Unclear | Yes | Yes | Yes | Yes | Unclear | Yes | 5 |
| Furusawa M | 2023 | Yes | Yes | Yes | Yes | Yes | Yes | Yes | Yes | 8 |
| Rostom AY | 2000 | Yes | Yes | Yes | Yes | Yes | Yes | Yes | Yes | 8 |
| Handy C | 2021 | Yes | Yes | Yes | Yes | Yes | Yes | Yes | Yes | 8 |
| Carrier X | 2020 | Yes | Yes | Yes | Yes | Yes | Yes | Yes | Yes | 8 |
| Emadzadeh A | 2025 | Yes | Yes | Yes | Yes | Yes | Yes | Yes | Yes | 8 |
| Parsi M | 2019 | Yes | Yes | Yes | Yes | Yes | Yes | Yes | Yes | 8 |

Scoring: Yes = clearly reported; No = not reported; Unclear = insufficient information. Total score out of 8. Abbreviations: JBI, Joanna Briggs Institute.
