# Supplementary material for: Tumor lysis syndrome in breast cancer: a systematic review of the literature
Source: Front Oncol. 2026 Oct 14;16:1937565. doi: 10.3389/fonc.2026.1937565 (PMC13713353; doi:10.3389/fonc.2026.1937565)
Supplement: Supplementary file 3 [file Table3.docx]

**Supplementary Table S3 – Individual patient-level laboratory and management data**

| First author | Year | Uric acid peak (mg/dL) | Potassium peak (mmol/L) | Phosphorus peak (mg/dL) | Calcium nadir (mg/dL) | LDH peak (U/L) | Creatinine peak (mg/dL) | Baseline renal function | TLS management | Outcome |
| --- | --- | --- | --- | --- | --- | --- | --- | --- | --- | --- |
| Kikuchi M | 2024 | 14.1 | 6.9 | 8.7 | 7 | 1904 | 2.64 | Cr 0.94 mg/dL | Hydration, febuxostat, hemodialysis | Died |
| Zigrossi P | 2001 | NR | NR | NR | NR | Elevated | NR | NR | Supportive care | Survived |
| Watkinson GE | 2021 | 12.4 (0.74 mmol/L) | 6.3 | 5.8 (1.87 mmol/L) | NR | NR | 3.21 (284 μmol/L) | eGFR 48 mL/min | IV fluids, rasburicase 3 mg × 3 days | Died |
| Tasnim N | 2026 | 12 | 6 | 13 | NR | 3052 | 3.6 | Cr 0.6 mg/dL | IV fluids, rasburicase, mechanical ventilation | Died |
| Taira F | 2015 | 22.2 | 8.1 | 6.4 | 7.4 | 2297 | 7.06 | Cr 0.64 mg/dL | Hydration, calcium gluconate, furosemide | Died |
| Baudon C | 2016 | 11.4 | 6.4 | 1.64 | 1.73 | 2473 | Elevated (GFR 38 mL/min) | eGFR 53 mL/min | IV fluids, vasopressors | Died |
| Nishimura K (1) | 2015 | Elevated | NR | Elevated | NR | Elevated | Elevated | NR | Hydration, urate-lowering therapy | Survived |
| Nishimura K (2) | 2015 | Elevated | NR | Elevated | NR | Elevated | Elevated | NR | Hydration, urate-lowering therapy | Survived |
| Aslam HM | 2019 | 18.2 | 5.4 | 6.7 | 9.6 | NR | 3.38 | eGFR 14 mL/min | IV fluids, allopurinol | Died |
| Kawaguchi Ushio A | 2013 | Elevated | NR | NR | NR | NR | Elevated | NR | Rasburicase | Survived |
| Vaidya GN | 2015 | 11.3 | 5.7 | 7.1 | NR | 9390 | 3.4 | Cr <1.0 mg/dL | IV fluids, rasburicase, hemodialysis | Died |
| Mott FE (1) | 2005 | 10 | 4.5 | 6.2 | 5.9 | 916 | 2.4 | Cr 2.4 mg/dL | Hydration, allopurinol | Survived |
| Mott FE (2) | 2005 | 15 | 6.3 | 8.9 | 6.5 | 3270 | 1.7 | Cr 0.6 mg/dL | Hydration, allopurinol, rasburicase | Survived |
| Kurt M | 2004 | NR | NR | NR | NR | NR | NR | NR | NR | Died |
| Furusawa M | 2023 | 8.5 | 7.7 | NR | NR | NR | 0.76 | Cr 0.45 mg/dL | IV fluids, hemodialysis | Survived |
| Rostom AY | 2000 | Elevated | Elevated | Elevated | Low | NR | Elevated | WBC 11.1, ESR 105 | IV fluids, allopurinol | Died |
| Handy C | 2021 | 16.2 | 8.2 | 8.2 | 8.5 | 226 | 15.15 | Cr 0.69 mg/dL | IV fluids, allopurinol, rasburicase | Survived |
| Carrier X | 2020 | 17.8 | 5.5 | 5 | 9.3 | NR | 4.5 | Cr 0.80 mg/dL | IV fluids, rasburicase | Survived |
| Emadzadeh A | 2025 | 13 | 7.6 | 14 | 6.8 | 1055 | 5.4 | NR | IV fluids, allopurinol, hemodialysis | Died |
| Parsi M | 2019 | 13.4 | 6.5 | 4.7 | NR | 937 | 1.4 | NR | IV fluids, rasburicase, allopurinol | Survived |
